# Hinokiflavone induces apoptosis via activating mitochondrial ROS/JNK/caspase pathway and inhibiting NF‐κB activity in hepatocellular carcinoma

**DOI:** 10.1111/jcmm.15474

**Published:** 2020-06-09

**Authors:** Wan Mu, Xuefang Cheng, Xue Zhang, Ying Liu, Qianzhou Lv, Gaolin Liu, Jigang Zhang, Xiaoyu Li

**Affiliations:** ^1^ Department of Pharmacy Shanghai Eye Diseases Prevention and Treatment Center/Shanghai Eye Hospital Shanghai General Hospital, National Clinical Research Center for Eye Diseases Shanghai Key Laboratory of Ocular Fundus Diseases Shanghai Engineering Center for Visual Science and Photomedicine Shanghai engineering research center of precise diagnosis and treatment of eye diseases Shanghai China; ^2^ Department of Clinical Pharmacy Shanghai General Hospital Shanghai Jiao Tong University School of Medicine Shanghai China; ^3^ Department of Pharmacy Zhongshan Hospital Fudan University Shanghai China

**Keywords:** apoptosis, G0, G1 cell cycle arrest, hepatocellular carcinoma, hinokiflavone, JNK, NF‐κB, ROS

## Abstract

Hepatocellular carcinoma (HCC) is the sixth most common malignancy with limited treatment options. Hinokiflavone (HF), a natural biflavonoid, has shown to inhibit the proliferation of melanoma, whereas its antitumour effect against HCC and the underlying mechanisms remain elusive. Here, we aimed at evaluating its antitumour effect against HCC in both in vitro and in vivo. Cell counting kit 8, colony formation assay, PI/RNase staining and Western blotting revealed that HF inhibited the proliferation of HCC cells via G0/G1 cell cycle arrest with p21/p53 up‐regulation. DAPI staining, Annexin V‐FITC/PI staining and Western blotting confirmed that HF triggered caspase‐dependent apoptosis. Moreover, HF increased the levels of mitochondrial reactive oxygen species (mtROS) and activated c‐Jun N‐terminal kinase (JNK) pathway, as measured by MitoSOX Red staining and Western blotting. After respectively inhibiting mtROS (Mito‐TEMPO) and JNK (SP600125), HF‐induced apoptosis was reversed. Additionally, Western blotting documented that HF suppressed nuclear factor kappa B (NF‐κB) activity and the anti‐apoptotic genes downstream, contributing to cell apoptosis. Finally, in vivo studies demonstrated that HF significantly impaired tumour growth in HCC xenograft. Collectively, these findings suggested that HF induced apoptosis through activating mtROS/JNK/caspase pathway and inhibiting NF‐κB signalling, which may represent a novel therapeutic agent for treating HCC.

## 1 INTRODUCTION

**FIGURE 1:**
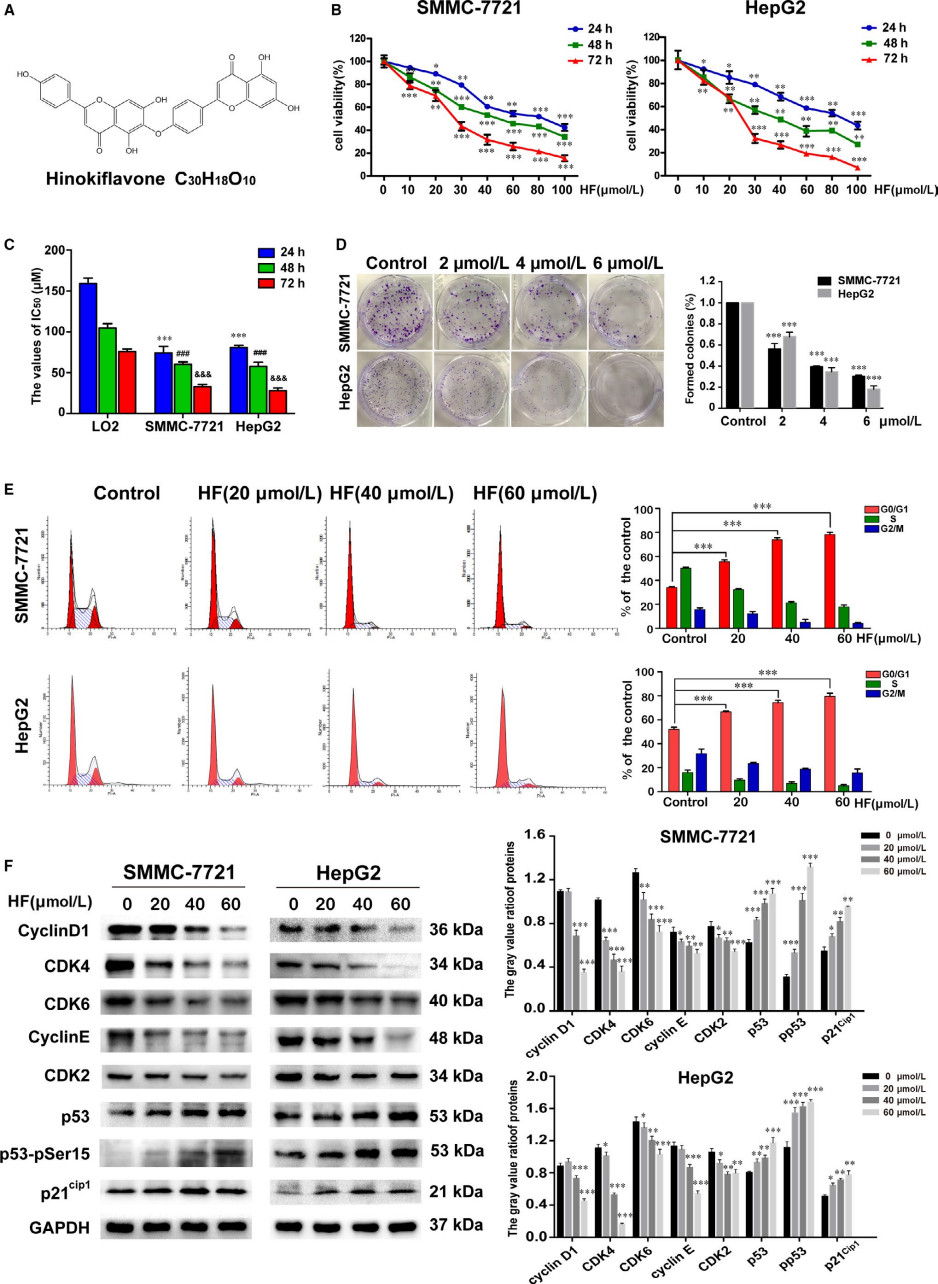
HF inhibits cell proliferation and induces G0/G1 phase cell cycle arrest in human HCC cells. (n = 3, $\bar{x}$ ± *s*). A, The chemical structure of Hinokiflavone (C_30_H_18_O_10_). B, The antitumour effect of HF on SMMC‐7721, HepG2 cells was measured with the CCK8 assay. *P < .05, **P < .01, ***P < .001 vs the control group. C, The IC_50_ values of HF for SMMC‐7721, HepG2 and LO2 cells, respectively in 24, 48 and 72 h. **P < .01 vs 24 h IC_50_ for LO2, ^###^ *P* < .001 vs 48 h IC_50_ for LO2, ^&&&^ *P* < .001 vs 72 h IC_50_ for LO2. D, Colony formation assay of SMMC‐7721 and HepG2 cells with control, HF. ***P < .001 vs the control group. E, The cell cycle of SMMC‐7721 and HepG2 cells exposed to HF (0, 20, 40 and 60 µmol/L) for 24 h were detected followed by flow cytometry assay. ***P < .001 vs G0/G1 phase of control. F, Western blotting was used to analyse the protein expression of G0/G1 phase‐related proteins. *P < .05, **P < .01, ***P < .001 vs the control group. 0.1% DMSO served as vehicle control or 0 µmol/L

Hepatocellular carcinoma (HCC) is the sixth most common cancers and a second leading cause of cancer‐related deaths worldwide.^1^ However, there has no effective chemotherapy for the treatment of HCC because of acquired chemoresistance.^2^ It is of great significance to screen novel antitumour compound to improve the survival rates in patients with HCC. Numerous herbal plant‐derived natural compounds occupy a very important position in the area of cancer chemotherapy.^3^ Naturally occurring biflavonoids belong to a subclass of the plant flavonoid family and consist of a dimer of flavonoids connected with C‐C or C‐O‐C bonds.^4^ Hinokiflavone (HF, Figure 1A), a biflavonoid with C‐O‐C connection, mainly isolated from *Selaginella tamariscina*, *Selaginella sinensis* and *Selaginella moellendorffii* which were of extremely strong drought resistance, and widely distributed in China.^5^ It was reported that HF would be metabolized through the rupture of ether linkage between two units of flavone, glutamine conjugation and glycine conjugation in vivo.^6^ The particular metabolic pathways may contribute to the extensive various pharmacological activities of HF, such as anti‐HIV‐1 activity,^7^ anti‐inflammatory activity ^8^ and antioxidant activity.^9^

Noteworthily, the anticancer activity of HF has attracted much attention.^10^, ^11^ Recent study has shown that HF can be acted as an inhibitor of mRNA spliceosomes in human cancer cells, interfering with the post‐transcriptional translation process and inducing tumour cell death.^12^ Moreover, the biflavonoid HF has been reported to induce apoptosis through the reactive oxygen species (ROS)‐mediated apoptosis in melanoma.^13^ However, most of pre‐clinical researches on the antitumour effect of HF have not clarified its specific targeted signalling pathways. Excessive amounts of ROS may trigger the activation of c‐Jun N‐terminal kinase (JNK), a stress‐activated protein kinase of the mitogen‐activated protein kinase (MAPK) family,^14^ which is a key modulator in mitochondria‐mediated intrinsic apoptotic pathways.^15^ In addition, pre‐clinical research indicated that the application of HF for the treatment of inflammatory bowel diseases, due to its potential role in the inhibition of nuclear factor kappa B (NF‐κB) and extracellular regulated kinase (ERK)1/2 signalling.^8^ Abrogation of NF‐κB activation is considered as an effective therapeutic approach for the prevention and treatment of human HCC.^16^ Thus, all these pre‐clinical researches indicate the huge potentiality of HF against HCC. Although most of pre‐clinical researches indicate the huge potentiality of HF for the development of new antitumour drugs, the anticancer effect for HCC is not very clear and needs more investigations.

Here, we first comprehensively investigated the potent antitumour efficacy of HF against human hepatoma cells and the underlying molecular mechanisms both in vitro and in vivo. We found that HF showed a strong growth inhibitory effect against two HCC cell lines (SMMC‐7721 and HepG2). Further study had proved that HF induced G0/G1 phase arrest and caspase‐dependent mitochondrial apoptosis. The mitochondrial ROS (mtROS)/JNK/caspase signalling pathway played a crucial role in HF‐induced apoptosis. Taken together, our discovery opens up a new source of potential therapeutic drugs for clinical treatment of HCC and explains the molecular mechanism behind HF.

## 2 MATERIALS AND METHODS

### 2.1 Reagents, chemicals and antibodies

Hinokiflavone (HF) with purity >98% was purchased from Chengdu Herb purify Co., Ltd., batch number: B‐051‐180730. A stock solution of HF at 200 mmol/L concentration was prepared in DMSO (Sigma) and stored at −20°C. Broad‐spectrum caspase inhibitor (z‐VAD‐FMK), caspase‐3 specific inhibitor (z‐DEVD‐FMK), caspase‐9 specific inhibitor (z‐LEHD‐FMK), JNK inhibitor (SP600125) and p38 inhibitor (SB202190) were procured from MedChemExpress. Dulbecco's modified Eagle's medium (DMEM), foetal bovine serum (FBS), penicillin‐streptomycin, phosphate buffered saline (PBS) and 0.25% trypsin were obtained from Gibco/BRL. The primary antibodies such as CDK4, CDK6, cyclin D1, CDK2, cyclin E, p21^Cip1^, p53, p‐p53 Ser15, cleaved caspase‐3, cleaved caspase‐9, cleaved poly (ADP‐ribose) polymerase (c‐PARP), B cell lymphoma 2 (Bcl‐2), Bcl‐2 associated x (Bax), cytochrome *c* (cyt *c*), p‐p38, p38, p‐JNK, JNK, c‐Jun, p‐IKBα, IKBα, p‐p65, p65 and glyceraldehyde‐3‐phosphate dehydrogenase (GAPDH) were provided by Cell Signaling Technology. The antibodies IAP1/2, XIAP were provided by Abcam, and c‐FLIP was provided by santa cruz biotechnology.

### 2.2 Cell culture

Human HCC cell line SMMC‐7721 and the normal hepatic cell line LO2 were gifted from the Institute of Clinical Translational Research, Shanghai General Hospital. HepG2 cell line was obtained from Cell Bank of Shanghai Institute of Biochemistry and Cell Biology, Chinese Academy of Sciences. All of these cells were cultured in high‐glucose DMEM supplemented with 10% FBS and 1% penicillin‐streptomycin (100 U/mL of penicillin and 100 µg/mL for streptomycin) and maintained at 37°C in a humidified atmosphere with 5% CO_2_. The cells of logarithmic growth period, which grow up to about 80%‐90% confluency in the culture dish, can be used for experiments.

### 2.3 Cell viability assay

Cell counting kit 8 (CCK8) assay (Dojindo) was used to measure anti‐proliferative effect of HF. Briefly, SMMC‐7721 and HepG2 cells cultured in 96‐well plates were treated with different concentrations of HF (0, 10, 20, 30, 40, 60, 80 and 100 µmol/L). LO2 cells were treated with different concentrations of HF (0, 10, 20, 40, 80, 120,160, 240 and 320 µmol/L). After 24, 48 or 72 hours, cells in each well were incubated with 10% CCK8 working solution for another 1 or 2 hours at 37°C. Absorbance was measured at 450 nm wavelength in each well using a microplate reader (Bio‐Tek). Data are represented as the mean of five replicates.

### 2.4 Cell cycle analysis

The effect of HF on cell cycle stages was analysed by flow cytometer (BD Accuri C6) with PI/RNase staining buffer (BD Biosciences). Briefly, cells at a density of 4 × 10^5^ cells per well were plated on six‐well plates and treated with increasing concentrations (0, 20, 40 and 60 µmol/L) of HF for 24 hours. After treatment, cells were harvested into single cell suspension and fixed in 500 µL of 70% cold ethanol at −20°C for at least 4 hours. Before flow cytometry analysis, cells were washed with PBS and stained with PI/RNase staining buffer for 15 minutes. The data were analysed with ModFit LT software (FACS Calibur).

### 2.5 Morphological evaluation

4′,6‐diamidino‐2‐phenylindole (DAPI) staining was performed to observe morphological characteristics of apoptotic cells. In brief, cells were exposed to HF for 48 hours and then fixed with 4% paraformaldehyde for 30 minutes. The cells were washed with PBS and treated with 100 µL of DAPI (Beyotime Biotechnology) for 10 minutes at room temperature in the dark. After washing twice with PBS, the cells were assessed under a fluorescence microscope (Leica) to observe nuclear fragmentation and chromatin condensation.

### 2.6 Apoptosis detection by flow cytometry

The percentage of apoptotic cells was assessed by flow cytometry using an Annexin V‐FITC/PI apoptosis detection kit (BD Pharmingen). Briefly, cells were seeded in six‐well plates at a density of 4 × 10^5^ cells per well, and the adherent cells were treated with HF (0, 20, 40 and 60 µmol/L) for 48 hours. Both floating and adherent cells were collected. After washing twice with cold PBS, cells were resuspended in 1× binding buffer and incubated with 5 µL of Annexin V‐FITC and 5 µL of PI for 15 minutes at room temperature in the dark. Samples were analysed by flow cytometry within 1 hour of the staining procedure.

### 2.7 Clone formation assay

The detailed methods of clone formation assay were in Appendix S1.

### 2.8 Measurement of MMP

Mitochondrial membrane potential (MMP, Δψm) was measured with the mitochondrion‐specific cationic fluorescence dye JC‐1 (Beyotime Biotechnology). See Appendix S1 for detailed methods.

### 2.9 Measurement of mitochondrial ROS

MitoSOX Red dye (Invitrogen Life Technologies, M36008) was used to determine the mtROS level. See Appendix S1 for detailed methods.

### 2.10 Western blot analysis

Western blot was performed using standard protocols. The detailed methods were described in Appendix S1.

### 2.11 Immunofluorescence and nuclear staining

Immunofluorescence was used to examine the changes in the subcellular localization of p65 after HF treatment. See Appendix S1 for detailed methods.

### 2.12 HCC xenograft experiment

Twenty‐four male BALB/c‐nu athymic mice (Shanghai SLAC Laboratory Animal Co., Ltd., Certificate) at 4 weeks of age were housed in a standard animal laboratory supplied with sterilized water and food. All animal‐related procedures were in strict accordance with the PR China legislation on the use and care of laboratory animals and were approved by the Animal Care and Use Committee of Shanghai General Hospital, Shanghai, China. The detailed methods of HCC xenograft and drug administration were described in Appendix S1. Tumour volume was measured every other 4 days with a sliding caliper until animals were killed to observe dynamic changes in tumour growth, and it was calculated by a standard formula as follows: length × width^2^/2. After completion of drug administration, all animals continued to be kept in normal feeding for another week, and then, the tumour tissues were excised from the euthanized mice, weighed and fixed in 5% formalin for immunohistochemical analysis, H&E staining and TUNEL assay.

### 2.13 Data statistics

All data were presented as mean ± standard deviation (mean ± SD). Statistical significances were determined by GraphPad Prism version 5 (GraphPad Software, Inc). Student's t test was used to compare the control and treatment groups, and multiple comparisons were performed using one‐way ANOVA. *P*‐values below .05 were considered statistically significant.

## 3 RESULTS

### 3.1 HF inhibits cell proliferation and induces G0/G1 cell cycle arrest in human HCC

Hinokiflavone (HF) significantly decreased the viability of SMMC‐7721 and HepG2 cells in a time‐ and dose‐dependent manner (Figure 1B). As shown in Figure 1C, IC_50_ values for SMMC‐7721 were 74.4 ± 8.1 µmol/L (24 hours), 60.3 ± 2.9 µmol/L (48 hours) and 33.0 ± 2.6 µmol/L (72 hours), while that for HepG2 were, respectively, 80.8 ± 2.6 µmol/L (24 hours), 57.5 ± 5.3 µmol/L (48 hours) and 28.1 ± 2.7 µmol/L (72 hours). As shown in Figure 1C and Figure S1, The IC_50_ values of HF for LO2 cells were 159.1 ± 5.6 µmol/L (24 hours),104.7 ± 4.5 µmol/L (48 hours) and 75.7 ± 2.8 µmol/L (72 hours), showing much lower cytotoxicity compared to HCC cells. Moreover, colony formation assay showed that the number of colonies formed of SMMC‐7721 and HepG2 cells significantly reduced with the increasing concentrations of HF (Figure 1D). These results confirmed the anti‐proliferation activity of HF against HCC cells. Additionally, the results of cell cycle analysis revealed that HF induced an increase in the percentage of cells in G0/G1 phase (Figure 1E), with down‐regulation of cyclin D1, cyclin‐dependent kinase 4 (CDK4), and cyclin‐dependent kinase 6 (CDK6) and up‐regulation of total p53 along with p‐p53 ser15 and p21^Cip1^ (Figure 1F). Taken together, these results showed that HF‐induced G0/G1 phase arrest may be accounted for the anti‐proliferative effect against HCC cells.

### 3.2 HF induces caspase‐dependent intrinsic apoptotic pathway in human HCC

**FIGURE 2:**
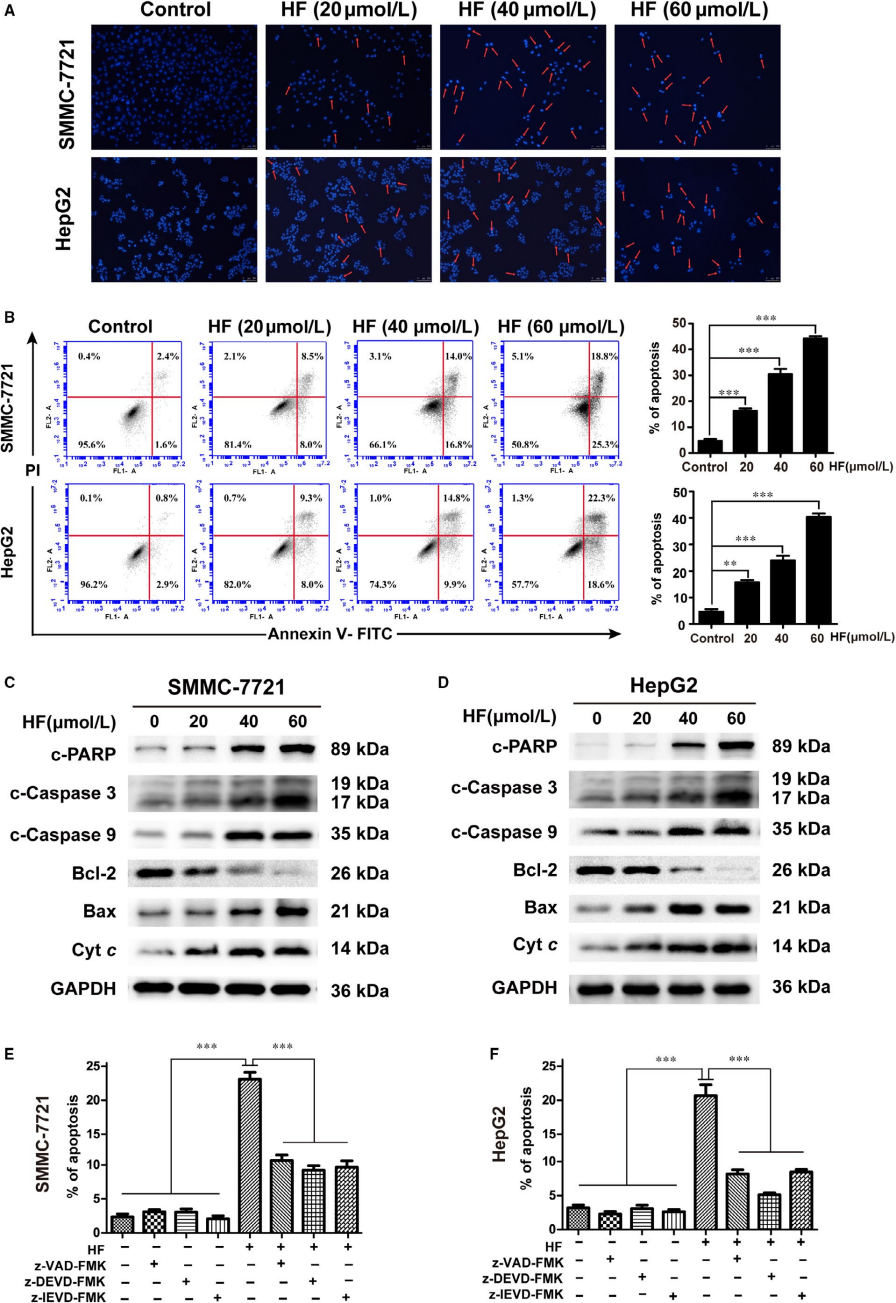
HF induces caspase‐dependent intrinsic apoptotic pathway in human HCC cells. (n = 3, $\bar{x}$ ± *s*). A, DAPI staining and fluorescence microscopy were used to detected apoptotic nuclear morphology induced by HF. Magnification of ×100, scale bars = 200 µm. Red arrows indicate chromatin and nuclear fragmentation. B, The apoptotic rates of HCC cells exposed to HF were measured and analysed by flow cytometry using Annexin V‐FITC/PI staining. **P < .01, ***P < .001 vs the control group. C, D, The expression levels of cleaved PARP, cleaved caspase‐3, cleaved caspase‐9, Bcl‐2, Bax and cyt *c* proteins were analysed by Western blotting. E, F, Annexin V‐FITC/PI staining and flow cytometry were used to detected the apoptotic rates of SMMC‐7721 and HepG2 pre‐incubated with z‐VAD‐FMK, Z‐DEVD‐FMK or Z‐LEHD‐FMK for 4 h, before treatment with HF. ***P < .001 vs the HF group

DAPI staining revealed that HF increased blue fluorescence intensity of DAPI of nucleus (Figure 2A), which indicated that HF may trigger different degrees of cell shrinkage, nuclear fragmentation and chromatin condensation. Annexin V‐FITC/PI double staining with flow cytometry analysis showed that the rate of early and late apoptotic cells increased after treatment with HF in a dose‐dependent manner for 48 hours (Figure 2B). In addition, the expression levels of cleaved PARP, cleaved caspase‐3 and cleaved caspase‐9 were increased in SMMC‐7721 and HepG2 (Figure 2C, D, Figure S2C). Furthermore, we pre‐treated HCC cells with a pan‐caspase inhibitor z‐VAD‐FMK (50 µmol/L), specific caspase‐3 inhibitor z‐DEVD‐FMK (100 µmol/L) and specific caspase 9 inhibitor z‐LEHD‐FMK (50 µmol/L) for 4 hours, followed by treatment with HF (40 µmol/L) for 24 hours to determine whether HF induced caspase‐dependent apoptosis. Our results show that Z‐VAD‐FMK, z‐DEVD‐FMK and z‐LEHD‐FMK respectively reduced the proportion of apoptosis in response to HF by 11.97%, 13.37% and 12.93% for SMMC‐7721, and by 11.53%, 14.57% and 11.23% for HepG2 (Figure 2E, F). These results suggested that HF may induce caspase‐dependent apoptosis in HCC.

Considering mitochondria play a key role in intrinsic apoptotic pathway. We used JC‐1, a mitochondrion‐specific fluorescent probe, to detect MMP (Δψm) with flow cytometry and fluorescence microscopy. Compared with the corresponding control, HF caused an obvious decrease of MMP (Δψm) as evident from the decrease in the intensity ratio of red fluorescence and green fluorescence in both HCC cells (Figure S2A, B). Moreover, with depolarization of mitochondria, there was a decrease in the expression ratio of mitochondria‐associated protein Bcl‐2/Bax and an increase in the accumulation of cyt *c* in the cytoplasm (Figure 2C, D, Figure S2D). Thus, HF induces caspase‐dependent apoptosis through activation of the inner mitochondria‐mediated apoptotic pathway.

### 3.3 HF‐induced apoptosis involves activation of JNK in human HCC

**FIGURE 3:**
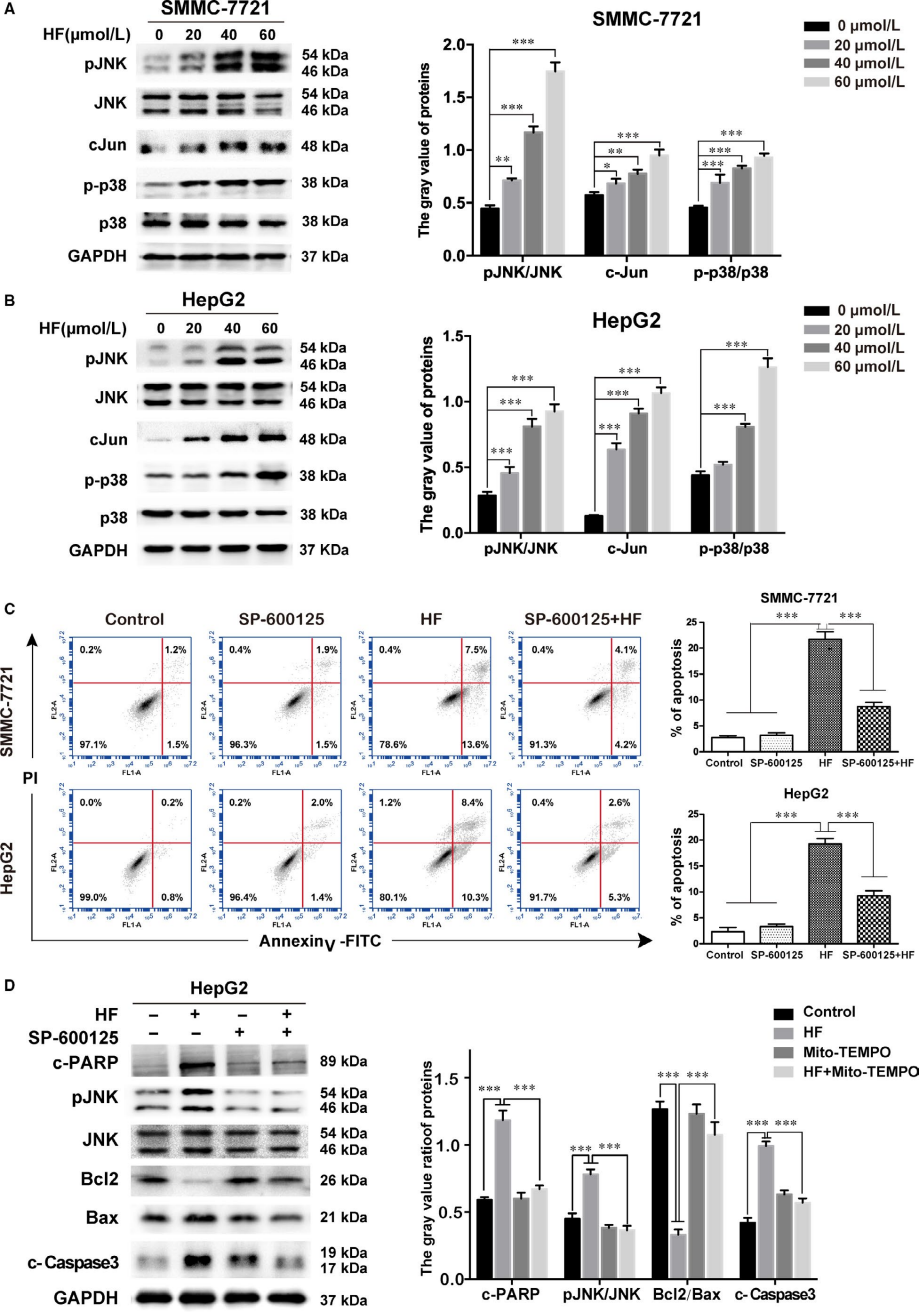
HF‐induced apoptosis involves JNK activation in human HCC cells. (n = 3, $\bar{x}$ ± *s*). A, B, The expressions of MAPKs proteins in SMMC‐7721 and HepG2 cells were analysed by Western blotting. *P < .05, **P < .01, ***P < .001 vs the control group. C, Annexin V‐FITC/PI staining and flow cytometry were used to detected the apoptotic rates of SMMC‐7721 and HepG2 pre‐incubated with SP600125 (10 µmol/L) for 3 h, before treatment with HF (40 µmol/L) for 24 h. ***P < .001 vs the HF group. D, HepG2 cells were given the same treatment in Figure 5C. The expression of p‐JNK, JNK, Bcl2, Bax, cleaved PARP and cleaved caspase‐3 was determined by Western blotting. ***P < .001 vs the HF group

Western blotting was used to examine the potential effect of HF on MAPK pathway. Interestingly, HF treatment induced the activation of JNK and p38 in a dose‐dependent manner (Figure 3A, B). To further test the role of JNK and p38 activation on HF‐induced apoptosis, HCC cells were pre‐treated with 10 µmol/L SP600125 (JNK inhibitor) or 20 µmol/L SB202190 (p38 inhibitor) for 3 hours, followed by treatment with 40 µmol/L HF for 24 hours. Addition of SP600125 remarkably reversed HF‐induced apoptosis by 13.00% in SMMC‐7721, and by 10.04% in HepG2 (Figure 3C), whereas there was on obvious effect on HF‐induced apoptosis in response to pre‐treatment with SB202190 (Figure S3).

Moreover, SP600125 attenuated the decrease of ratio of Bcl/Bax protein expression in response to HF as well as resulted in reduction of cleaved caspase‐3 and cleaved PARP (Figure 3D). These results indicated the activation of JNK plays a critical role in HF‐induced caspase‐dependent apoptosis in HCC cells.

### 3.4 MtROS production contributes to JNK‐mediated caspase‐dependent apoptosis induced by HF in human HCC

**FIGURE 4:**
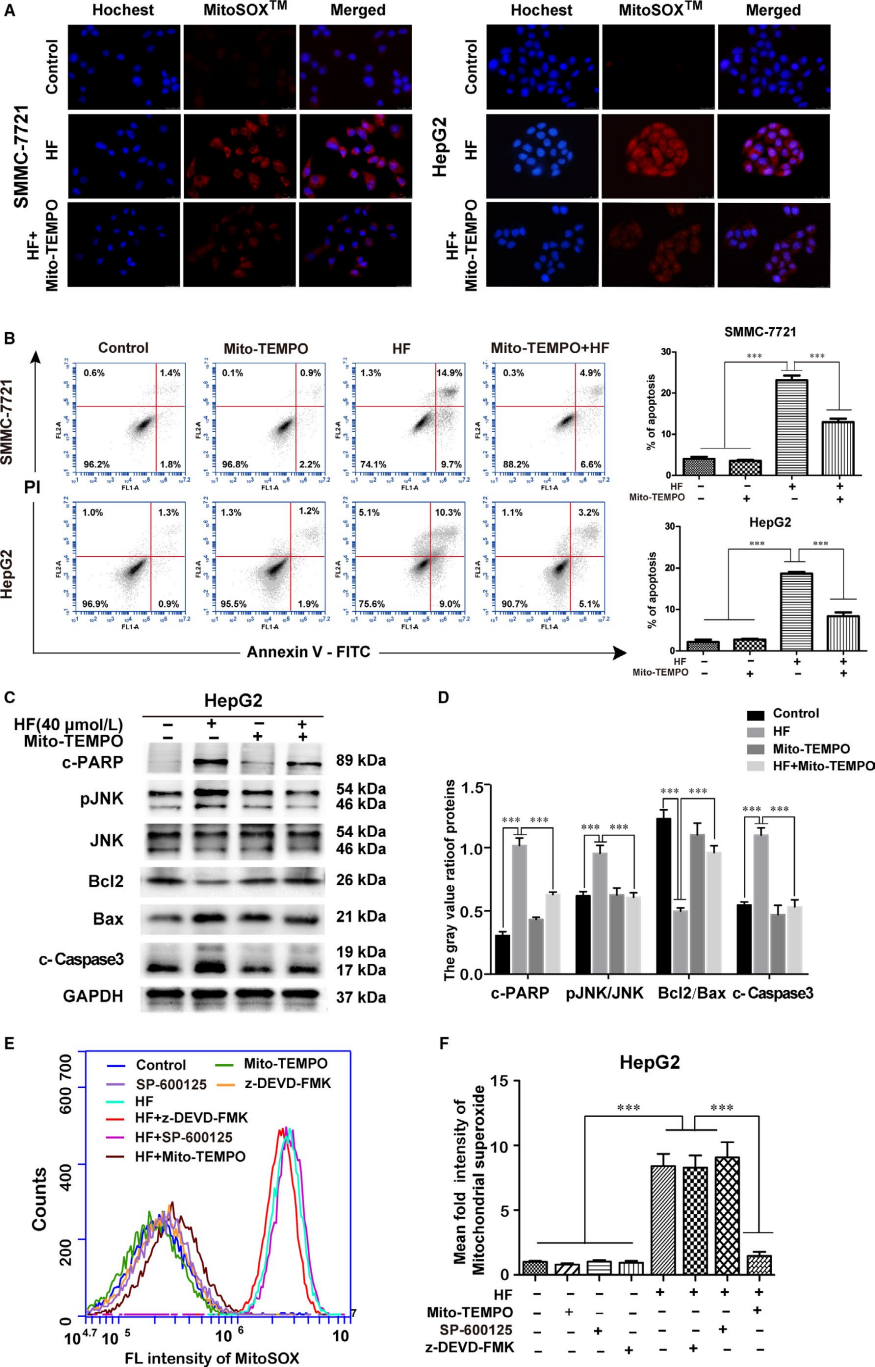
MtROS contributes to JNK‐mediated caspase‐dependent apoptosis induced by HF. (n = 3, $\bar{x}$ ± *s*). A, MitoSOX Red dye staining and fluorescence microscope were used to determine the level of mitochondrial ROS of HCC cells treated with HF in the presence or absence of Mito‐TEMPO. Magnification of 400×, scale bars = 25 µm. B, Annexin V‐FITC/PI staining and flow cytometry were used to detected the apoptotic rates of SMMC‐7721and HepG2 pre‐incubated with Mito‐TEMPO for 4 h, before treatment with HF. ***P < .001 vs the HF group. C, D, HepG2 cells were given the same treatment in Figure 6B. Expression of p‐JNK, JNK, Bcl2, Bax, cleaved PARP and cleaved caspase‐3 was determined by Western blotting. ***P < .001 vs the HF group. E, F, MitoSOX Red dye staining and flow cytometry were used to detect the mitochondrial ROS of HepG2 pre‐incubated with SP600125, z‐DEVD‐FMK or Mito‐TEMPO for 4 h, before treatment with HF. Statistical analysis of the mean fluorescence intensity in F. ***P < .001 vs the HF group

Intracellular ROS is an important regulator of apoptosis induction.^17^ Mitochondrion is regarded as the major source and target of ROS, which contributes to activate caspase‐dependent apoptosis.^18^ MitoSOX Red (a novel mtROS specific probe) was used to target the detection of ROS superoxide radicals in the mitochondria. Treatment with HF resulted in an increase in the fluorescence intensity of MitoSOX Red in both HCC cells, whereas the increased fluorescence intensity attenuated in response to mitochondrion‐targeted triphenylphosphonium‐conjugated antioxidant (Mito‐TEMPO, a specific mitochondrion‐targeting antioxidant, Sigma‐Aldrich) (Figure 4A). Moreover, pre‐treatment of antioxidant Mito‐TEMPO (100 µmol/L) respectively reversed the HF‐induced apoptosis proportion by 10.17% in SMMC‐7721, and by 10.23% in HepG2 cells (Figure 4B). In addition, Mito‐TEMPO almost blocked HF‐induced the prolonged activation of JNK, decrease of Bcl‐2/Bax ratio, activation of caspase‐3 and cleavage of PARP1 (Figure 4C, D). Moreover, pre‐treatment of cells with Mito‐TEMPO significantly reduced the ROS production induced by HF, whereas ROS level remained unchanged in response to pre‐treatment with SP600125 or z‐DEVD‐FMK (Figure 4E, F). These results proved that HF‐induced mtROS production contributed to activate JNK signalling, which further triggered the caspase‐dependent apoptosis in human HCC.

### 3.5 HF‐induced apoptosis is mediated through the inhibition of NF‐κB signalling in human HCC

**FIGURE 5:**
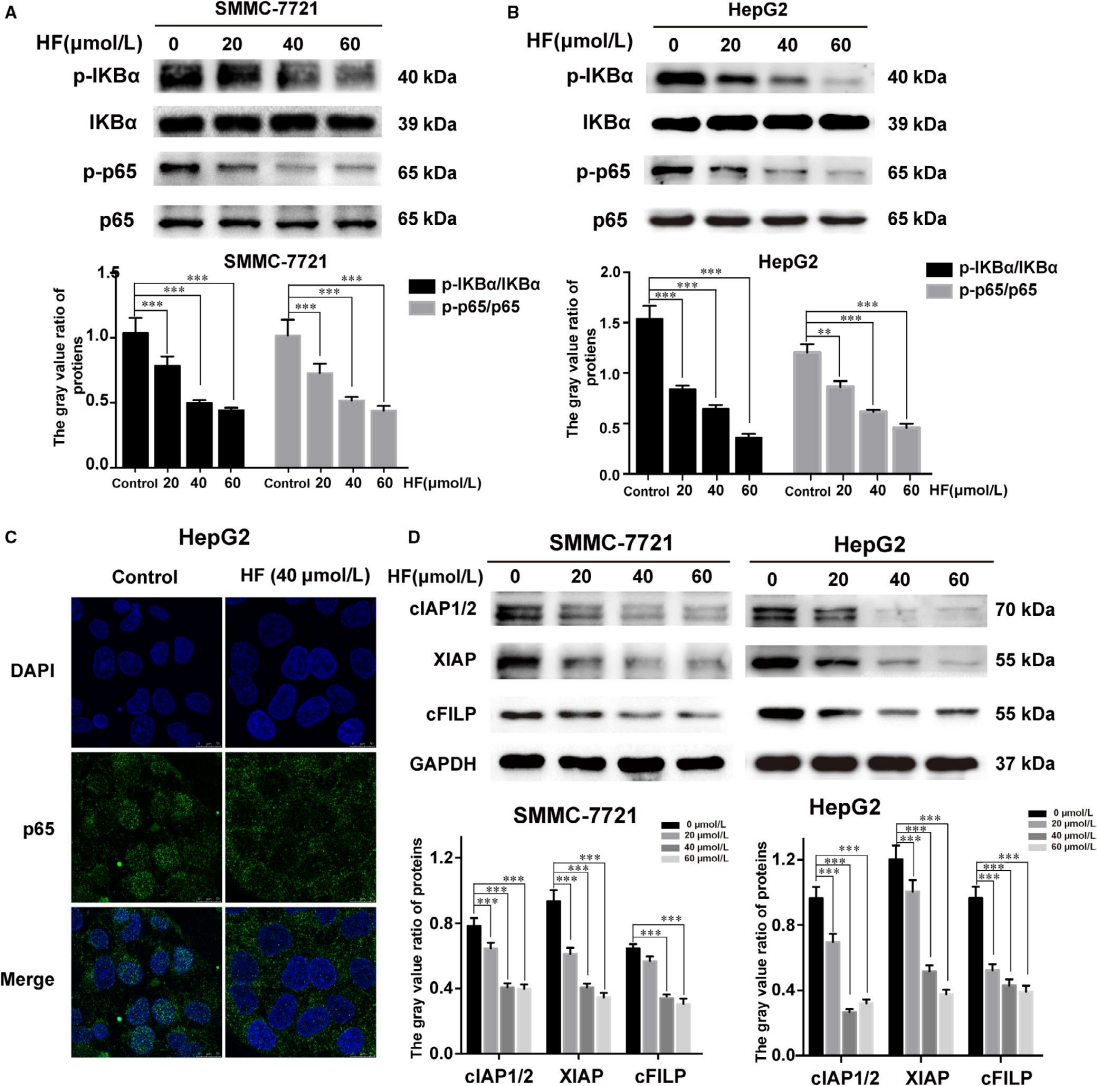
HF‐induced apoptosis involves the inhibition of NF‐κB activity in human HCC cells. (n = 3, $\bar{x}$ ± *s*). A, B, Western blot analysis demonstrates the levels of p‐IKBα, IKBα, p‐p65 and p65 in SMMC‐7721 and HepG2 cells treated with HF. Mean optical density of p‐IKBα/IKBα and p‐p65/p65 was quantified by Image‐Pro Plus. **P < .01, ***P < .001 vs control. C, For assessing nuclear translocation of p65, immunofluorescence microscopy was performed to examine the translocation of NF‐κB/p65 subunit, as described in section of Materials and Methods. Magnification, 630×. Scale bar, 50 µm. D, The expression levels of cIAP1/2, XIAP and cFILP were examined by Western blot analysis. Each experiment was performed in triplicates. *P < .05, **P < .01, ***P < .01 vs control

Studies show that NF‐κB is a heterodimer transcription factor consisting of p50 and p65, and the expression of p65 in human HCC tissues is much higher than that in the surrounding non‐tumour liver tissue.^19^ To detect whether HF could attenuate NF‐κB/p65 activity in HCC, the expression of p‐IKBα, IKBα, p‐p65 and p65 after treatment with various concentrations of HF (20, 40 and 60 µmol/L) for 48 hours was detected with Western blotting. It was shown that HF suppressed the phosphorylation of IKBα and p65 in HCC cells in a dose‐dependent manner (Figure 5A, B). The fluorescence images of p65 shown in Figure 5C revealed that HF induced a substantial decrease in the nuclear localization of p65 in HepG2 cells. The expression levels of several anti‐apoptosis‐related NF‐κB target genes (cIAP1/2, XIAP and c‐FLIP) were remarkably decreased after treatment with HF (Figure 5D). These data suggest that HF‐induced apoptosis involves the inhibition of NF‐κB activation through the suppression of IKBα phosphorylation and p65 nuclear translocation in HCC cell lines.

### 3.6 HF inhibits tumorigenesis of HCC xenograft in vivo

**FIGURE 6:**
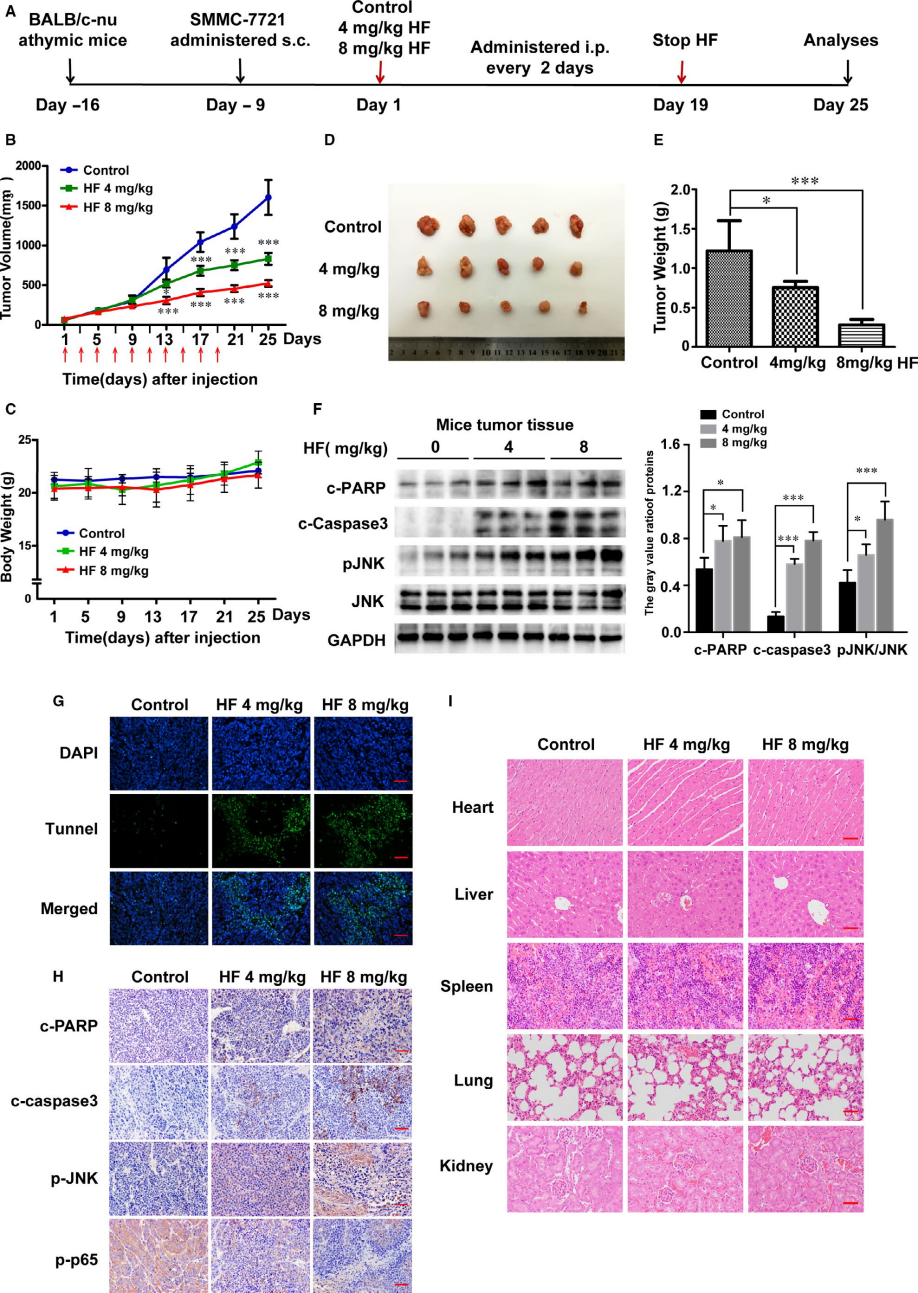
HF inhibits tumorigenesis of HCC xenograft in vivo. (n = 5, $\bar{x}$ ± *s*). A, BALB/c‐nu mice bearing SMMC‐7721 xenografts were intraperitoneally treated with 10% DMSO (negative control) or HF (4 or 8 mg/kg) every other day for ten times. B, C, Tumour sizes and bodyweights were measured every fourth day for 25 days. *P < .05, ***P < .001 vs the control group. D, Photographs of the resulting tumours excised from HCC subcutaneous xenografts. E, Statistical analysis of the tumour weights. *P < .05, ***P < .001 vs the control group. F, The level of cleaved PARP, cleaved caspase‐3, p‐JNK and JNK in tumour xenograft tissues was detected by Western blotting. **P < .01, ***P < .001 vs the control group. G, The apoptotic status in tumour tissues was assessed with TUNEL assay. Magnification of 200×, scale bars = 50 µm. H, H&E staining was used for histological analysis. The expression levels of cleaved PARP, cleaved caspase‐3 and p‐JNK were examined by immunohistochemistry. Magnification of 400×, scale bars = 25 µm. I, Tissue sections of heart, livers, lungs and kidneys of HF‐treated nude mice, as determined by H&E staining. Magnification of 400×. scale bars = 25 µm

To determine the anticancer effect of HF on HCC in vivo, HCC xenografts were established by subcutaneously transplanting SMMC‐7721 cells into the right flank of BALB/c‐nu mice. Eighteen mice were randomly divided into three groups (control, 4 mg/kg HF, 8 mg/kg HF) until tumour reached about 50 mm^3^, and administered i.p. every other day for ten times (Figure 6A). One mouse of the control group died of fighting, without any anatomical and pathological abnormalities by autopsy. HF significantly inhibited the growth of tumour (Figure 6B, D), but had no significant effect on bodyweight (Figure 6C). The average size (or weight) of the tumours in HF 4 mg/kg, and HF 8 mg/kg group was reduced to 831.8 mm^3^ (or 0.76 g) and 523 mm^3^ (or 0.28 g), compared to 1604 mm^3^ (or 1.22 g) in control group (Figure 6B, E). Furthermore, HF up‐regulated the expression levels of cleaved PARP, cleaved caspase‐3 and p‐JNK (Figure 6F). Terminal deoxynucleotidyl transferase dUTP nick‐end labelling (TUNEL)‐positive cells significantly increased in HF group, consistent with the results of in vitro studies (Figure 6G). Immunohistochemistry of HF‐treated tumour tissue demonstrated positive expression of cleaved PARP, cleaved caspase‐3 and p‐JNK (Figure 6H). Haematoxylin and eosin (H&E) staining of the excised organs revealed no alteration in the histological pattern for heart, liver, spleen, lungs and kidneys in all groups, suggesting that HF had no major organ‐related toxicity (Figure 6I). Above results demonstrate that HF inhibits the growth of human HCC in vivo.

## 4 DISCUSSION

Numerous reports have highlighted the potent antitumour activities of natural active compounds for cancer chemoprevention and treatment.^3^ Our present study clearly demonstrated HF inhibits the proliferation of human HCC cells, such as SMMC‐7721 and HepG2, but has low cytotoxicity against normal human hepatocyte LO2 cells, suggesting a selective antitumour action of HF to some degree (Figure 1B, C). Moreover, we further comprehensively investigated the detailed molecular mechanism for anticancer effects of HF on HCC both in vitro and in vivo.

Deregulation of cell cycle regulation is an important hallmark of tumour progression. Cell cycle arrest followed by apoptosis induction may be an effective strategy of anticancer drugs against uncontrolled proliferation of cancer cells.^20^ The results of cell cycle analysis with flow cytometry showed that HF increased the proportion of cells in G0/G1 phase (Figure 1F). The progression of eukaryotic cell cycle is controlled through various CDK complexes via phosphorylation of downstream target proteins. Cyclin D1‐CDK4/6 complexes play important roles in the progression of cell entry in G1 phase, whereas the CDK2‐cyclin E complex is essential for the transition from G0/G1 to S cell phase.^21^, ^22^ Consistent with the flow cytometry results, HF significantly reduced the expression levels of G0/G1 phase‐related regulatory proteins (including CDK4, CDK6, cyclin D1, cyclin E and CDK2)(Figure 1F). It has reported that the p53 tumour suppressor can be activated by phosphorylation at ser15 (various stress stimuli response), which can coordinates an adaptive gene expression programme of P21^Cip1^, leading to growth arrest or cell death. As P21^Cip1^ negatively regulate the activity of cyclin D1‐CDK4/6 and cyclin E‐CDK2 complexes, thereby inducing G0/G1 cell cycle arrest.^21^, ^23^, ^24^ Interestingly, we found increased expression levels of total p53 along with p‐p53 ser15 and p21^Cip1^ after treatment with HF in HCC cells (Figure 1F). Thus, these results demonstrate that HF induces the G0/G1 phase arrest through activating the p53/p21^Cip1^ signal pathway and decreasing the expression of G0/G1 phase‐related cell cycle regulatory proteins in HCC cells.

Apoptosis is a critical type of cell death process activated by various chemotherapeutic agents against human cancers,^25^ which is characterized with cell membrane blebbing, cell shrinkage, chromatin condensation and nuclear fragmentation.^26^ At molecular levels, apoptosis is mainly regulated by the cell surface death receptor‐directed extrinsic apoptotic pathway and the mitochondria‐mediated intrinsic apoptotic pathway,^27^ which both activate the aspartate‐specific cysteine protease (caspase) cascade, ultimately resulting in the cleavage of caspase‐3 and critical cellular proteins (PARP).^28^ Caspase‐9 is an important intracellular amplifier of caspase signalling downstream of mitochondria in the intrinsic apoptosis pathway. Our results revealed that HF triggered the activation of caspase‐9, caspase‐3 and subsequent cleavage of PARP1 (Figure 2C, D), as well as the result that the caspase inhibitor reversed HF‐induced apoptosis in SMMC‐7721 and HepG2 cells (Figure 2E, F), suggesting that HF could induce caspase‐dependent apoptosis in HCC cells. Moreover, the increased level of cleaved caspase‐3 was also confirmed in HF‐treated tumour tissues with Western blot and immunohistochemical analysis (Figure 6F, H). The intrinsic apoptotic pathway can be activated by expression of several mitochondrial molecular targets, such as B cell lymphoma 2 family (Bcl‐2, Bax), cytochrome *c* (cyt *c*) and caspases in response to cellular stress, hypoxia and ROS.^29^ The transition of reduced expression ratio of Bcl‐2 and Bax proteins (Bcl‐2/Bax) leads to a sharp decrease in mitochondrial membrane potential, which subsequently contribute to cyt *c* release from the mitochondrial intramembrane space to the cytosol, thereby activating the cytosolic caspases.^30^ In the present study, we detected the decrease in the ratio of Bcl‐2/Bax, as well as an increase in the accumulation of cyt *c* in HCC cells exposed to HF (Figure 2C, D, and Figure S2D). The decrease in MMP induced by HF was also well observed (Figure S2A, B). Thus, HF‐induced cell apoptosis may be mediated through the intrinsic mitochondrion‐mediated apoptotic pathway.

The mitogen‐activated protein kinases (MAPKs) family, including extracellular regulated kinase (ERK) 1/2, JNK and p38, are mediators of cellular responses to extracellular signals. JNK and p38 MAPKs closely associated with cell apoptosis, which can be induced by chemical‐triggered stress responses.^31^, ^32^ To investigate the upstream pathways involved in HF‐induced cell death, the effects of HF on MAPK activation were examined. We found that HF induced a sustained activation of JNK and p38 phosphorylation in SMMC‐7721 and HepG2 cells (Figure 3A, B). A large number of literature have proved that the active JNK signalling could initiate the mitochondrion‐derived apoptosis via modulation of the expression of pro‐ or anti‐apoptotic proteins (Bax and Bcl‐2) translocated onto mitochondria.^15^, ^33^ Interestingly, we found that the inhibition of JNK with SP600125 significantly reversed HF‐induced apoptosis (Figure 3C), and attenuated the decrease of ratio of Bcl/Bax protein expression, activation of caspase‐3 and cleavage of PARP1 in response to HF (Figure 3D). These results indicate the activation of JNK trigger HF‐induced caspase‐dependent mitochondrial apoptosis in HCC.

ROS, as an active form of oxygen, is derived from NADPH oxidase 3 (NOX3) and mitochondrion during cellular metabolism.^18^, ^34^ Oxidative stress, caused by intracellular excessive amounts of ROS, is an important regulator of mitochondrion‐mediated apoptotic pathways.^35^, ^36^ Our study shown that ROS presumably generated through mitochondria accumulated after HF treatment (Figure 4A); the blocking of mtROS accumulation with Mito‐TEMPO almost completely reversed HF‐induced apoptosis (Figure 4B). The inhibition of mtROS with Mito‐TEMPO almost blocked HF‐induced the prolonged activation of JNK, decrease of Bcl‐2, increase of Bax and activation of caspase‐3, and cleavage of PARP1 (Figure 4C, D). Furthermore, the pre‐treatment of cells with Mito‐TEMPO significantly alleviated the generation of ROS; however, the JNK inhibitor (SP600125) and caspase 3 inhibitor (z‐DEVD‐FMK) failed to induce change in the increased level of ROS in response to HF, indicating that ROS production occurs upstream of JNK and caspase activation (Figure 4E, F). Taken together, we illustrate that HF triggered apoptosis of HCC cells through the activation of caspase‐dependent pathway mediated via the mtROS/JNK/caspase pathway.

It is known that targeted suppression of NF‐κB activity can disrupt the anti‐apoptotic functions and proliferative activity of HCC cells, which has been regarded as a useful cancer therapeutic approach to prevent the progression of HCC carcinogenesis.^16^ Interestingly, our investigation indicated that HF treatment remarkably inhibited NF‐κB activity through the suppression of IKBα phosphorylation and p65 nuclear translocation in HCC cell lines (Figure 5A–C). cIAP1/2, XIAP and c‐FLIP are the transcription and expression products of NF‐κB target genes that contribute to the cell proliferation and the resistance to apoptosis by suppressing caspase activation.^37^ Consistently, the expression of IAP1/2, XIAP and c‐FLIP were significantly down‐regulated following HF treatment in HCC cell lines (Figure 5D). All these results supported our hypothesis that HF‐induced apoptosis partly associated with the inhibition of NF‐κB activity in HCC. Additionally, cumulative evidences have suggested the suppression of NF‐κB pathway increases the level of intracellular ROS by up‐regulating expression of antioxidant proteins such as Cu‐Zn‐superoxide dismutase, Mn‐superoxide dismutase and glutathione S transferase Pi.^34^ Thus, we still need further investigation to elucidate the correlation between the suppression of NF‐κB and the accumulation of ROS in response to HF, which will provide a more accurate explanation to the anticancer efficacy of HF for HCC.

**FIGURE 7:**
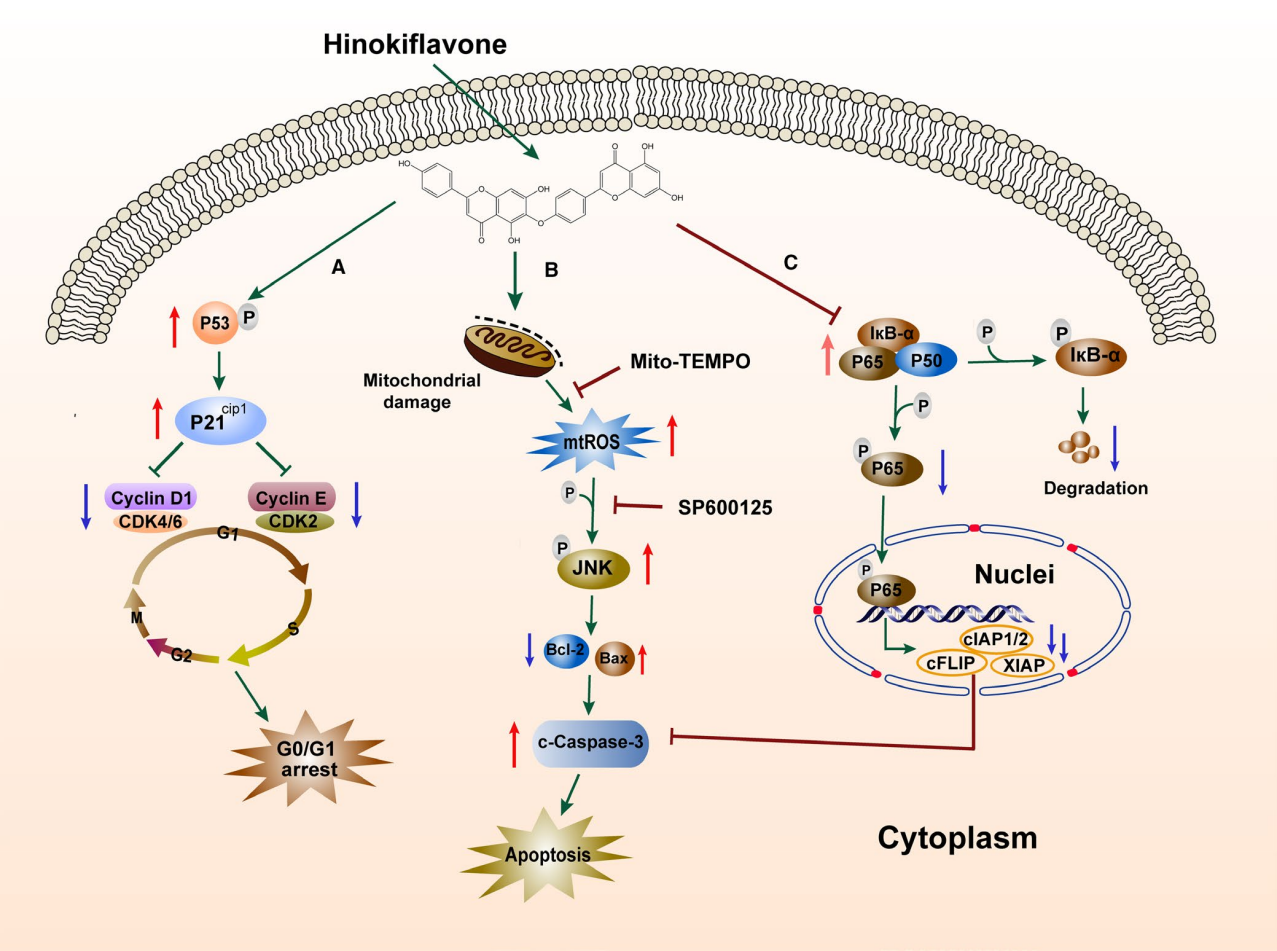
Potential anticancer mechanism of HF for HCC. A, Schematic showing the anticancer mechanism for hinokiflavone in hepatocellular carcinoma (HCC) cells. Hinokiflavone (HF) suppressed cell proliferation by G0/G1 cell cycle arrest through up‐regulating the expression level of p53, p‐p53 ser15 and p21^Cip1^ proteins and decreasing the activation of G0/G1 phase‐related cell cycle regulatory proteins. B, HF triggered the increased production of mitochondrial reactive oxygen species (mtROS), which activated c‐Jun N‐terminal kinase (JNK) pathway, and subsequently attenuated the decrease of ratio of Bcl/Bax protein expression, further contributing to the caspase‐dependent mitochondrial apoptosis. C, HF significantly inhibited the activation of nuclear factor kappa B (NF‐κB) signalling, which contributed to caspase‐dependent apoptosis through suppressing the expression of NF‐κB target anti‐apoptotic genes, including cellular inhibitor of apoptosis 1 and 2 (cIAP1/2), X‐linked inhibitor of apoptosis (XIAP) and cellular FLICE‐inhibitory protein (c‐FLIP)

In conclusion, we demonstrated the antitumour effects of HF on HCC cells in vitro and in vivo, and confirmed that HF significantly suppressed cell proliferation by G0/G1 cell cycle arrest and subsequently induced cell apoptosis through the activation of mtROS/JNK/caspase‐mediated intrinsic apoptotic pathways and the inhibition of NF‐κB signalling pathway in HCC. Additionally, we also showed that HF decreased tumour growth in BALB/c‐nu mice bearing HCC cell xenografts (Figure 6). Histopathological data showed no significant histological alterations in the heart, liver, lungs and kidneys from BALB/c‐nu mice treated with HF, indicating that HF was well‐tolerated and induced no obvious systemic toxicity in the tested animals. Overall, our study provides an insight into the molecular mechanism underlying HF‐induced cell death and may help in the development of a potentially safe agent against HCC. This provides an overview of mechanism diagram for anticancer effect of HF on human HCC in Figure 7.

## CONFLICT OF INTEREST

The authors declare that no conflict of interest.

## AUTHOR CONTRIBUTION

**Wan Mu:** Conceptualization (lead); Data curation (lead); Formal analysis (lead); Investigation (lead); Methodology (lead); Project administration (lead); Validation (lead); Visualization (lead); Writing‐original draft (lead); Writing‐review & editing (equal). **Xuefang Cheng:** Investigation (supporting); Methodology (equal); Project administration (supporting); Resources (equal); Software (equal); Visualization (supporting); Writing‐original draft (supporting); Writing‐review & editing (equal). **Xue Zhang:** Investigation (supporting); Methodology (supporting); Project administration (supporting); Resources (equal); Software (equal). **Ying Liu:** Data curation (equal); Resources (equal); Software (equal); Validation (equal); Writing‐review & editing (equal). **Qianzhou Lv:** Formal analysis (supporting); Software (supporting); Validation (supporting). **Gao‐Lin Liu:** Formal analysis (supporting); Resources (supporting); Software (supporting); Validation (supporting). **Jigang Zhang:** Conceptualization (supporting); Data curation (supporting); Formal analysis (equal); Funding acquisition (supporting); Supervision (supporting); Validation (supporting); Writing‐review & editing (equal). **Xiaoyu Li:** Conceptualization (equal); Funding acquisition (lead); Investigation (supporting); Supervision (lead); Visualization (lead); Writing‐review & editing (equal).

## Supporting information

App S1

## Data Availability

The data that support the findings of this study are available from the corresponding author upon reasonable request.

## References

[1] Torre LA , Bray F , Siegel RL , et al. Global cancer statistics, 2012. CA Cancer J Clin. 2015;65:87‐108. 25651787 10.3322/caac.21262

[2] Marin JJG , Briz O , Herraez E , et al. Molecular bases of the poor response of liver cancer to chemotherapy. Clin Res Hepatol Gastroenterol. 2018;42:182‐192. 29544679 10.1016/j.clinre.2017.12.006

[3] Atanasov AG , Waltenberger B , Pferschy‐Wenzig EM , et al. Discovery and resupply of pharmacologically active plant‐derived natural products: a review. Biotechnol Adv. 2015;33:1582‐1614. 26281720 10.1016/j.biotechadv.2015.08.001 PMC4748402

[4] Gontijo VS , Dos Santos MH , Viegas C Jr . Biological and chemical aspects of natural biflavonoids from plants: a brief review. Mini Rev Med Chem. 2017;17:834‐862. 27823559 10.2174/1389557517666161104130026

[5] Yin R , Xiong K , Wen S , et al. Development and validation of an LC‐MS/MS method for the determination of hinokiflavone in rat plasma and its application to a pharmacokinetic study. Biomed Chromatogr. 2017;31:e3821‐e3826. 10.1002/bmc.3821 27552190

[6] Chen Y , Feng X , Li L , et al. UHPLC‐Q‐TOF‐MS/MS method based on four‐step strategy for metabolites of hinokiflavone in vivo and in vitro. J Pharm Biomed Anal. 2019;169:19‐29. 30831449 10.1016/j.jpba.2019.02.034

[7] Lin YM , Anderson H , Flavin MT , et al. In vitro anti‐HIV activity of biflavonoids isolated from Rhus succedanea and Garcinia multiflora. J Nat Prod. 1997;60:884‐888. 9322359 10.1021/np9700275

[8] Shim SY , Lee SG , Lee M . Biflavonoids isolated from selaginella tamariscina and their anti‐inflammatory activities via ERK 1/2 signaling. Molecules. 2018;23:e926. 29673161 10.3390/molecules23040926 PMC6017943

[9] Wang G , Yao S , Zhang XX , Song H . Rapid screening and structural characterization of antioxidants from the extract of selaginella doederleinii hieron with DPPH‐UPLC‐Q‐TOF/MS method. Int J Anal Chem. 2015;2015:849769. 25792983 10.1155/2015/849769 PMC4352518

[10] Lee IS , Nishikawa A , Furukawa F , et al. Effects of Selaginella tamariscina on in vitro tumor cell growth, p53 expression, G1 arrest and in vivo gastric cell proliferation. Cancer Lett. 1999;144:93‐99. 10503882 10.1016/s0304-3835(99)00202-5

[11] Lin YM , Chen FC , Lee KH . Hinokiflavone, a cytotoxic principle from Rhus succedanea and the cytotoxicity of the related biflavonoids. Planta Med. 1989;55:166‐168. 2526343 10.1055/s-2006-961914

[12] Pawellek A , Ryder U , Tammsalu T , et al. Characterisation of the biflavonoid hinokiflavone as a pre‐mRNA splicing modulator that inhibits SENP. eLife. 2017;6:e27402. 28884683 10.7554/eLife.27402 PMC5619949

[13] Yang S , Zhang Y , Luo Y , et al. Hinokiflavone induces apoptosis in melanoma cells through the ROS‐mitochondrial apoptotic pathway and impairs cell migration and invasion. Biomed Pharmacother. 2018;103:101‐110. 29635122 10.1016/j.biopha.2018.02.076

[14] Kamata H , Honda S , Maeda S , et al. Reactive oxygen species promote TNFalpha‐induced death and sustained JNK activation by inhibiting MAP kinase phosphatases. Cell. 2005;120:649‐661. 15766528 10.1016/j.cell.2004.12.041

[15] Schroeter H , Boyd CS , Ahmed R , et al. c‐Jun N‐terminal kinase (JNK)‐mediated modulation of brain mitochondria function: new target proteins for JNK signalling in mitochondrion‐dependent apoptosis. Biochem J. 2003;372:359‐369. 12614194 10.1042/BJ20030201 PMC1223409

[16] Luedde T , Schwabe RF . NF‐kappaB in the liver–linking injury, fibrosis and hepatocellular carcinoma. Nat Rev Gastroenterol Hepatol. 2011;8:108‐118. 21293511 10.1038/nrgastro.2010.213 PMC3295539

[17] Chauhan D , Li G , Sattler M , et al. Superoxide‐dependent and ‐independent mitochondrial signaling during apoptosis in multiple myeloma cells. Oncogene. 2003;22:6296‐6300. 13679868 10.1038/sj.onc.1206734

[18] Simon HU , Haj‐Yehia A , Levi‐Schaffer F . Role of reactive oxygen species (ROS) in apoptosis induction. Apoptosis. 2000;5:415‐418. 11256882 10.1023/a:1009616228304

[19] Qiao L , Zhang H , Yu J , et al. Constitutive activation of NF‐kappaB in human hepatocellular carcinoma: evidence of a cytoprotective role. Hum Gene Ther. 2006;17:280‐290. 16544977 10.1089/hum.2006.17.280

[20] Pavletich NP . Mechanisms of cyclin‐dependent kinase regulation: structures of Cdks, their cyclin activators, and Cip and INK4 inhibitors. J Mol Biol. 1999;287:821‐828. 10222191 10.1006/jmbi.1999.2640

[21] Vermeulen K , Van Bockstaele DR , Berneman ZN . The cell cycle: a review of regulation, deregulation and therapeutic targets in cancer. Cell Prolif. 2003;36:131‐149. 12814430 10.1046/j.1365-2184.2003.00266.x PMC6496723

[22] Saleh AM , Aljada A , El‐Abadelah MM , et al. The anticancer activity of the substituted pyridone‐annelated isoindigo (5'‐Cl) involves G0/G1 cell cycle arrest and inactivation of CDKs in the promyelocytic leukemia cell line HL‐60. Cell Physiol Biochem. 2015;35:1943‐1957. 25870953 10.1159/000374003

[23] Gulappa T , Reddy RS , Suman S , et al. Molecular interplay between cdk4 and p21 dictates G0/G1 cell cycle arrest in prostate cancer cells. Cancer Lett. 2013;337:177‐183. 23684928 10.1016/j.canlet.2013.05.014 PMC3752915

[24] Bartek J , Lukas J . Mammalian G1‐ and S‐phase checkpoints in response to DNA damage. Curr Opin Cell Biol. 2001;13:738‐747. 11698191 10.1016/s0955-0674(00)00280-5

[25] Fesik SW . Promoting apoptosis as a strategy for cancer drug discovery. Nat Rev Cancer. 2005;5:876‐885. 16239906 10.1038/nrc1736

[26] Kroemer G , Galluzzi L , Vandenabeele P , et al. Classification of cell death: recommendations of the Nomenclature Committee on Cell Death 2009. Cell Death Differ. 2009;16:3‐11. 18846107 10.1038/cdd.2008.150 PMC2744427

[27] Zimmermann KC , Bonzon C , Green DR . The machinery of programmed cell death. Pharmacol Ther. 2001;92:57‐70. 11750036 10.1016/s0163-7258(01)00159-0

[28] Degterev A , Boyce M , Yuan J . A decade of caspases. Oncogene. 2003;22:8543‐8567. 14634618 10.1038/sj.onc.1207107

[29] Wu J , Zhao Y , Park YK , et al. Loss of PDK4 switches the hepatic NF‐kappaB/TNF pathway from pro‐survival to pro‐apoptosis. Hepatology. 2018;68(3):1111–1124. 29603325 10.1002/hep.29902 PMC6165716

[30] Krick S , Platoshyn O , McDaniel SS , et al. Augmented K(+) currents and mitochondrial membrane depolarization in pulmonary artery myocyte apoptosis. Am J Physiol Lung Cell Mol Physiol. 2001;281:L887‐L894. 11557592 10.1152/ajplung.2001.281.4.L887

[31] Kyriakis JM , Banerjee P , Nikolakaki E , et al. The stress‐activated protein kinase subfamily of c‐Jun kinases. Nature. 1994;369:156‐160. 8177321 10.1038/369156a0

[32] Shen HM , Liu ZG . JNK signaling pathway is a key modulator in cell death mediated by reactive oxygen and nitrogen species. Free Radic Biol Med. 2006;40:928‐939. 16540388 10.1016/j.freeradbiomed.2005.10.056

[33] Guo C , Yang M , Jing L , et al. Amorphous silica nanoparticles trigger vascular endothelial cell injury through apoptosis and autophagy via reactive oxygen species‐mediated MAPK/Bcl‐2 and PI3K/Akt/mTOR signaling. Int J Nanomedicine. 2016;11:5257‐5276. 27785026 10.2147/IJN.S112030 PMC5066858

[34] Zhang J , Wang X , Vikash V , et al. ROS and ROS‐mediated cellular signaling. Oxid Med Cell Longev. 2016;2016:4350965. 26998193 10.1155/2016/4350965 PMC4779832

[35] Lee CY , Su CH , Tsai PK , et al. Cadmium nitrate‐induced neuronal apoptosis is protected by N‐acetyl‐l‐cysteine via reducing reactive oxygen species generation and mitochondria dysfunction. Biomed Pharmacother. 2018;108:448‐456. 30241048 10.1016/j.biopha.2018.09.054

[36] Yang YJ , Baek JY , Goo J , et al. Effective killing of cancer cells through ROS‐mediated mechanisms by AMRI‐59 targeting peroxiredoxin I. Antioxid Redox Signal. 2016;24:453‐469. 26528922 10.1089/ars.2014.6187

[37] Amit S , Ben‐Neriah Y . NF‐kappaB activation in cancer: a challenge for ubiquitination‐ and proteasome‐based therapeutic approach. Semin Cancer Biol. 2003;13:15‐28. 12507553 10.1016/s1044-579x(02)00096-2

