# Supplementary material for: Hinokiflavone induces apoptosis via activating mitochondrial ROS/JNK/caspase pathway and inhibiting NF‐κB activity in hepatocellular carcinoma
Source: J Cell Mol Med. 2020 Jun 9;24(14):8151–65. doi: 10.1111/jcmm.15474 (PMC7348176; doi:10.1111/jcmm.15474)
Supplement: Supplementary file 1 — App S1 [file JCMM-24-8151-s001.docx]

**Appendix S1**

**Materials and Methods**

**1. Clone formation assay**

Cells at a density of 900 cells per well were seeded in six-well plates. After attachment, cells were treated with HF at different concentrations (0, 2, 4, and 6 μM) for 10 days until they grew into visible colonies. The medium was discarded and the cells were washed twice with PBS. After being fixed with 4% paraformaldehyde for 30 min, the cells were washed with PBS and the colonies were stained with 0.1% crystal violet for 10 min at 25°C room temperature. The clones with more than 50 cells were counted and the images were recorded using a digital camera.

**2. Measurement of MMP**

Briefly, cells were seeded in six-well plates at a density of 4 ×10^5^ cells per well and treated with HF (0, 20, 40, and 60 μM) for 9 h. To quantitatively assess MMP, cells were collected and suspended in 1×JC-1 staining buffer (Beyotime Biotechnology, Suzhou, China) for 30 min at 37°C in the dark. The stained cells were washed twice and analyzed with flow cytometer; the change in MMP was calculated as the fold change in green fluorescence intensity of the test as compared to that of control. To evaluate morphological changes associated with MMP, cells in six-well plates were washed twice with PBS and incubated with JC-1 at 37°C for 20 min in the dark. After washing twice with PBS, cells were incubated in DMEM. The images of the stained cells were obtained with a fluorescence microscope (Leica, Wetzlar, Germany) to determine the changes in MMP.

**3. Measurement of mitochondrial ROS**

Cells seeded in six-well plates (4×10^5^ cells per well) with different experimental treatment. Then cells were preincubated with Hoechst 33342 for 10 min, stained with MitoSOX Red dye (5 μM) at 37 °C in the dark for 10 min, and washed twice with PBS. Images were captured using a fluorescence microscope. In addition, cells are collected and resuspended in PBS, and mtROS level was quantitatively determined by flow cytometer.

**4. Western blot analysis**

Cells and tissues samples were lysed in a modified radioimmunoprecipitation assay (RIPA) buffer containing phenylmethanesulfonyl fluoride (Beyotime Biotechnology) for 30 min on ice. The protein concentration was determined by BCA protein assay kit (Beyotime Biotechnology) according to the manufacturer’s instruction. 20-40 μg of proteins from each sample were separated on 10-15% sodium dodecyl sulfate polyacrylamide gel electrophoresis (SDS-PAGE) and transferred onto polyvinylidene difluoride membranes (Millipore, Billerica, MA, USA). The membranes were blocked with 5% non-fat dry milk in TBST for 1.5h at room temperature, followed by overnight incubation with the appropriate primary antibodies at 4°C. After washing with TBST, secondary antibody conjugated with horseradish peroxidase was added and the blots were incubated at room temperature for 1.5h. Immuno-reactive bands were visualized using enhanced chemiluminescence reagent, and quantified by densitometry using software Image Lab 3.0 (Bio-Rad, USA). Protein expression levels were normalized to that of GAPDH, and presented as fold changes relative to the control.

**5. Immunofluorescence and nuclear staining**

After treatment with HF (0, 20, 40, and 60 μM) for 24 h, cells were washed in ice-cold PBS, fixed with 3.7% paraformaldehyde for 15 min, and permeabilized with 0.5%-1% Triton X-100 for 30 min at room temperature. The fixed cells were washed with PBS and blocked with 1% bovine serum albumin (BSA) for 30 min, and then overnight incubated with anti-p65 antibody at 4°C; the nuclei were stained with DAPI solution, and the antibody was detected using anti-rabbit IgG conjugated with Texas Red (Molecular Probes). Immunofluorescence images were evaluated under a confocal laser scanning microscope (Leica, Germany).

**6. HCC** **xenograft experiment**

After one-week acclimation, a total of 1×10^7^ SMMC-7721 cells suspended in 100 μL of cold PBS were subcutaneously administrated into the right flank of each mouse. Eighteen model mice were selected and randomized into three groups (control, 4 mg/kg HF, 8 mg/kg HF) when tumors reached a mean group size of approximately 50 mm^3^. HF dissolved in 10%DMSO/40% polyethylene glycol-400 (PEG-400)/PBS solution (1:4:5, volume) was administered intraperitoneally (4 or 8 mg/kg i.p.) to athymic mice every other day for 19 days. The negative control mice were intraperitoneally injected with 10% DMSO/40%PEG-400/PBS solution.


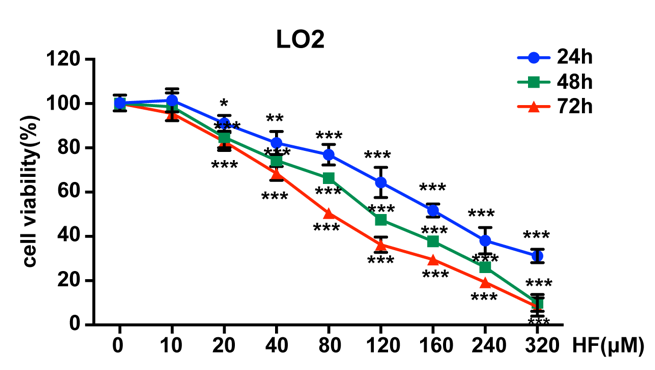


**Supplemental Figure 1.** The IC_50_ values of HF for LO2 cells, respectively in 24 h, 48 h and 72 h. *P < 0.05, **P < 0.01, ***P < 0.001 versus the control group. HF-induced apoptosis involves mitochondrion-mediated apoptotic pathway in human HCC cells.


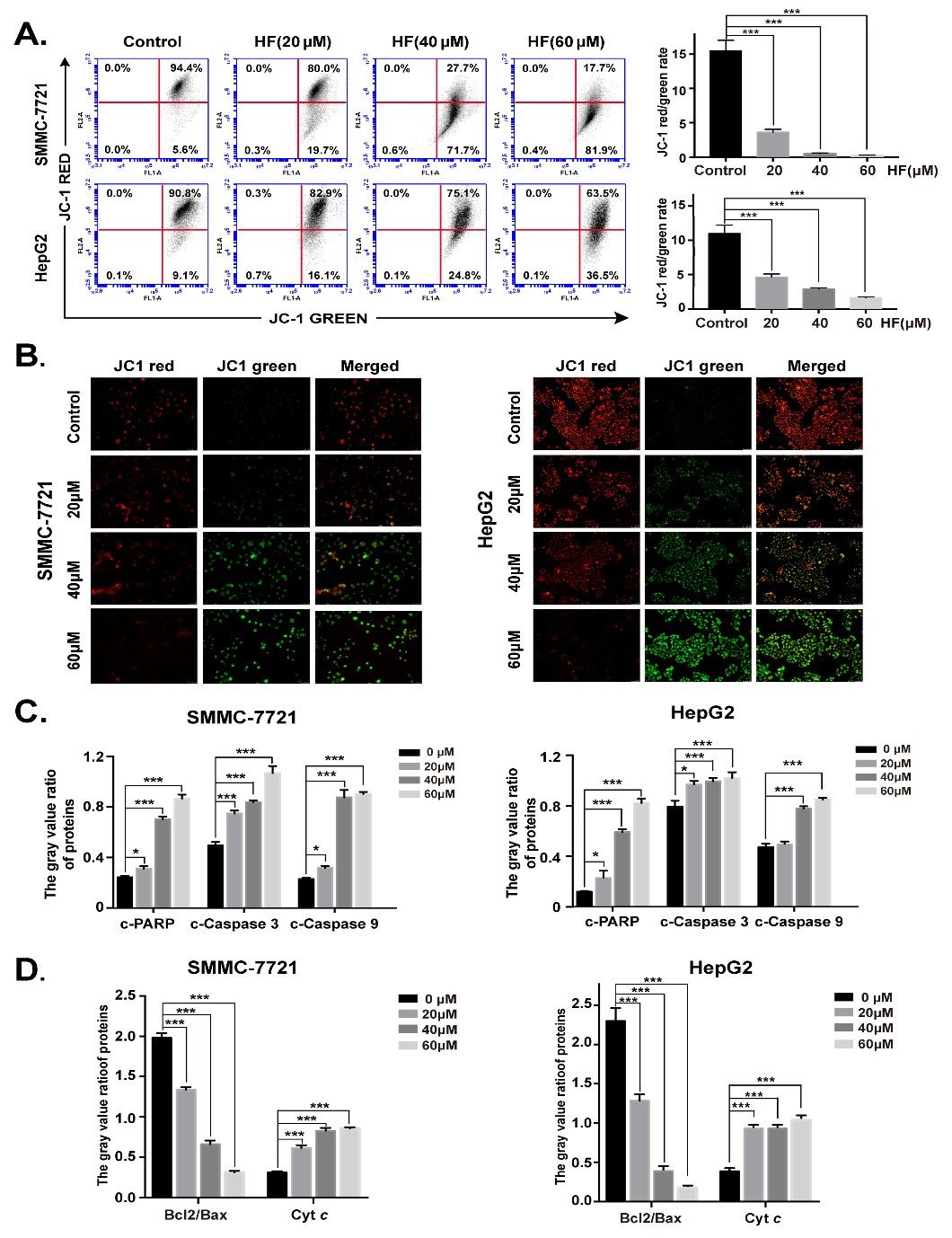


**Supplemental Figure 2.** HF-induced apoptosis involves mitochondrion-mediated apoptotic pathway in human HCC cells. (n=3, $\bar{\boldsymbol{x}}$±s)

The effect of HF on MMP was measured with the fluorescent mitochondrial probe JC-1. (A) Flow cytometry detection of JC1 staining in HCC cells, indicative of the HF-induced concentration-dependent decrease in MMP. ***P < 0.001 versus the control group. (B) Microscopic images detection of JC1 staining in SMMC-7721 and HepG2 cells, indicative of the HF-induced evident shift from the red to green in fluorescence. Magnification of 200×, scale bars = 50μm. (C, D) The statistical graphs of western blotting of Figure 2C, D. ImageJ software was used to quantify the relative gray value of protein expression. *P < 0.05, ***P < 0.001 versus the control group n=3.


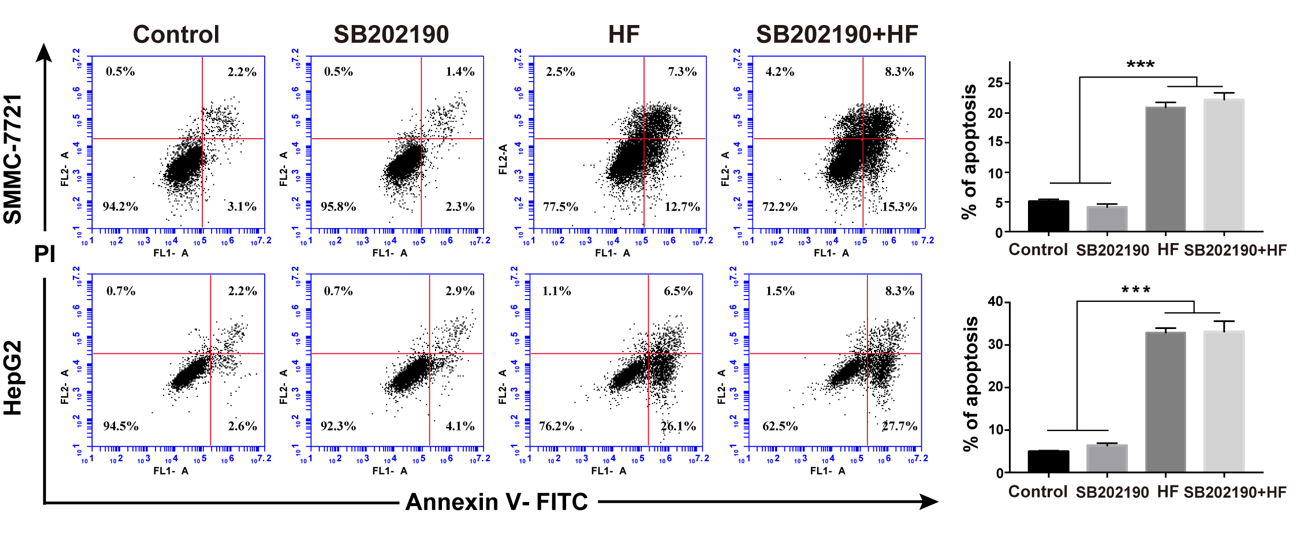


**Supplemental Figure 3.** The effect of p38 on HF-induced apoptosis in human HCC cells. (n=3, $\bar{\boldsymbol{x}}$±s) Annexin V-FITC/PI staining and flow cytometry were used to detected the apoptotic rates of SMMC-7721 and HepG2 pre-incubated with p38 inhibitor (SB202190, 20 μM) for 3 h, before treatment with HF (40 μM) for 24 h. ***P < 0.001 versus the HF group.
